# Round the Hospitals

**Published:** 1920-10-16

**Authors:** 


### ROUND THE HOSPITALS.

WE note that two of the successful candidates for the College scholarships have chosen the career of sister-tutor and two the public health course at King's College for Women arranged for health visitors. We rejoice to see the College taking an active part in the very important business of evolving the highest type of health visitor. The searching comments often made by those dissatisfied with nurses as health visitors should open the eyes of matrons to the fact that the ordinary course of training lacks something which it is not easy to supply later on. Perhaps the effect of concentrating the mind for three or four years too exclusively on the interests of the institution is cramping. But it is certain that the defects in hospital training which tend to mar the ripening health visitor ought to be detected and exposed. There is no better road to this than to take women who have proved their efficiency in the hospital wards and open out to them a course of public health study such as is offered at the King's College for Women under Dr. Lane-Claypon. The international character now imparted to this course is a very valuable asset.

IN Ireland the Minister of Health is the Chief Secretary, and he is responsible for the rules and regulations for enrolment on the State Register. These will doubtless correspond in general terms with those adopted for England and Scotland. A delicate situation, however, is pretty sure to arise in connection with the employment of nuns as nurses. In certain instances, as in Belfast, the nuns are trained nurses, and do their duty as such. But in others, which are in a considerable majority, the qualifications of the nuns are vague, indefinite, and unsatisfactory. Some of them may have done a year's hospital duty—others may not. They decline to perform all the duties of a nurse, including night duty, but they take precedence over the lay nurses both in status and in remuneration. This attitude is strongly resented by all nurses, Catholic and Protestant. But Boards of Guardians who appoint them make no secret of their preference. They prefer the nuns, because they do not "grouse." The question that will arise is whether a nun's qualifications will be subjected to impartial examination, and whether those whose training is inadequate will be disqualified. The Council will find themselves in a rather tight corner. Boards of Guardians are quite equal to flouting any decision arrived at by a Council appointed as a result of an Act of the Westminster Parliament. On the other hand, if the Council shows laxity where professional qualifications are in question it will fall into contempt amongst those whose support matters most.

BEST congratulations to the nurses of the Royal Infirmary, Sheffield, who, under the will of Lieutenant-Colonel John Sinclair White, C.B.E., M.D., of Dunluce, Bournemouth, become his residuary legatees after the death of his wife, to whom the property is left on trust for her life. The late physician was a well-known figure in Sheffield, where he spent the greater part of his life in the practice of his profession. It was intimate knowledge of the Royal Infirmary nurses which prompted him to dispose his large fortune, amounting to over £48,000, for the founding of a home of rest for their benefit. No surer evidence can be given of the honour in which nurses are held by those with whom they work in close association than is afforded by such deeds.

NEW documents relating to "The Case of Miss Cavell" have been thrown into the form of a book by Ambroise Got, their interpreter, who states that they were brought to him by a "German political personage." The documents disclose more fully than has ever before come to light the iniquitous character of the trial and the extent to which the proceedings were hurried on and the evidence garbled in order that no reprieve might be possible. The account of the execution is entirely different from anything hitherto published:—"Miss Edith Cavell was brought by the soldiers from a neighbouring house. Her eyes had been bandaged and her head covered with a black veil. Until this she had walked bravely, but now, when she realised that she was before the platoon of execution, her strength failed her. She swayed, and fell about twenty yards from the wall against which she was to stand. The German officer in command of the firing approached Miss Cavell, stretched on the ground, drew from his belt his regulation wide-mouthed revolver, knelt down, and, taking steady aim, fired." A correspondent acquainted with the circumstances suggests in the *Daily Telegraph* that the cause of Miss Cavell's faint was the sight of M. Baney being shot before her eyes.

SIR ARTHUR STANLEY speaks in the warmest terms of the enthusiasm for voluntary work which has made itself evident in the counties. It will be interesting to judge by the record of this week's experiences whether this enthusiasm will react for good on the hospitals and provoke a revival in the recruiting of probationers. We believe that wherever the matter is wisely handled the effect of Red Cross zeal must be a quickening of vocation, which will move many to offer themselves for training as nurses. As an aid to drawing women to understand the advantages of hospital training we commend an admirable little pamphlet issued by the authorities of the Middlesex Hospital to all who write for papers. It gives good illustrations of the Nurses' Homes, all the information required by the candidates, and a list of the appointments and public services open to the trained nurse. The separate Night Nurses' home at the Middlesex is an excellent feature, with its own entrance and sitting-room for night nurses. Such booklets relating to local hospitals ought to be available for distribution at all Red Cross gatherings.

**ROUND THE HOSPITALS**—*(continued)*.

It is not usual to take general interest in the report of the Chief Inspector of Factories and Workshops, but our readers may find in that recently issued for 1919 a good deal which bears on their own work. The effects of the general shortening of hours of work are beginning to be very conspicuous. It is solely when production depends on the speed of the machinery that the output is reduced in proportion nearly corresponding to the reduction of hours. Where output is largely or entirely dependent upon the exertion of the worker there is frequently no loss in production, and sometimes a great gain, through shortening the hours. The effect of shorter hours on the operatives has been found to be uniformly good, and the number of absentees has lessened to a surprising extent—in one instance from 40 to 10 per cent. Great difficulty is being experienced in regard to the supply of skilled women, manufacturers being unable to fill vacancies even by advertising and offering substantial inducements. The supply of women is, in fact, as short in manufacturing circles as in institutions.

---

Miss M. E. Johnston, R.R.C., has resigned her post as Matron to the Ulster Volunteer Force Hospital, Belfast, in order to take up a similar appointment in London. Many thousands of wounded and invalided patients passed through the wards during the four years of her office, and she is much regretted by nurses, sisters, and patients alike.

---

Dr. Brodie O'Connor, Assistant M.O.H. at Plymouth, received a very cordial farewell on her departure from Plymouth, after four years' work, to take up an appointment in London. The staff of the Public Health Department, including health visitors, met at the Town Hall, and presented her with a gold pendant set with aquamarine and pearls in a case with suitable inscription. Dr. Hall spoke most warmly of Dr. O'Connor's services to Plymouth, where on her arrival there was but one welfare centre with a small attendance, while now, owing to her exertions, there are four more centres and a daily attendance of ninety-eight mothers.

---

The Lord Mayor and Lady Mayoress have arranged a meeting at the Mansion House at 3 p.m. on St. Luke's Day, October 18, to bring the needs of the Great Northern Central Hospital before the public. The aim is to secure an extra £8,000 before the end of the year, and we publish the Lord Mayor's letter on p. 63.

---

An appeal has been made for flannel bed-jackets and nightgowns of the same material for the little in-patients at the Queen's Hospital for Children, Bethnal Green. Some 1,700 children pass through the wards every year out of the 40,000 cases dealt with, and the wear and tear of the garments for the 170 occupants of the beds may be readily imagined. Ages vary from birth to fourteen years, so kind donors cannot go far wrong in the size of their gifts.

---

The Nurses' Club, established under the auspices of the Northumberland and Durham branch of the College of Nursing, at 17 Windsor Terrace, Newcastle, was formally opened on September 29 by Lady Armstrong. Mr. Francis Priestman, who presided, said he had never known a fund more popular than that raised for the formation of the club, the first of its kind to be opened in the North of England. Nurses are in their element when acting as pioneers, and the College members in all parts of England are playing their part well as scions of the most original and lively society of women in existence.

---

The absurdity of the existing system under which in some localities public-health duties are sub-divided among various nurses was brought into the open at a discussion which took place recently at the meeting of the Children's Care Committee for Halstead and Belchamp districts. The Committee has two nurses working in the district; one has the care of children until they are five, and then the other nurse takes them over until they leave school. A third local nurse attends in the place. A recent instance was reported to the Committee, when at one house before 11 a.m. the three nurses had made separate calls to make reports on the condition of children. The mother "took the matter very nicely," and was, in fact, a good deal amused. The Committee wisely decided that it was no joke, and have informed the Essex Education Committee and the Essex County Council that in their opinion the Notification of Births Act and the Maternity and Child-Welfare Act ought to be administered by the County Council, and nurses appointed for the combined duties with reasonable areas. The Chairman, in strongly supporting the resolution, alluded to there being a certain amount of jealousy somewhere on the part of the old committees, who did not like to give up the work, but declared the thing to be primarily considered was the health of the children, and that it was desirable that one nurse should attend to the whole family.

---

Lambeth Board of Guardians have again considered the question as to whether they should pay their nursing staff overtime, in view of the fact that they are unable to obtain sufficient nurses to carry into effect their resolution that a forty-eight hours' working week shall be enforced. The local branch of the Poor-Law Workers' Trade Union forwarded a letter urging the guardians to pay for overtime, but the guardians decided that they could not do so. Application is again to be made to the Ministry of Health to sanction the scheme of non-resident probationer nurses, but it is doubtful if Dr. Addison will accede to the request in view of the strong attitude he has hitherto taken up against the innovation.

